# Ascaroside Expression in *Caenorhabditis elegans* Is Strongly Dependent on Diet and Developmental Stage

**DOI:** 10.1371/journal.pone.0017804

**Published:** 2011-03-15

**Authors:** Fatma Kaplan, Jagan Srinivasan, Parag Mahanti, Ramadan Ajredini, Omer Durak, Rathika Nimalendran, Paul W. Sternberg, Peter E. A. Teal, Frank C. Schroeder, Arthur S. Edison, Hans T. Alborn

**Affiliations:** 1 Center for Medical, Agricultural and Veterinary Entomology, USDA-ARS, Gainesville, Florida, United States of America; 2 Medical Institute and Biology Division, California Institute of Technology, Pasadena, California, United States of America; 3 Boyce Thompson Institute and Department of Chemistry and Chemical Biology, Cornell University, Ithaca, New York, United States of America; 4 Department of Biochemistry and Molecular Biology, High Magnetic Field Laboratory, University of Florida, Gainesville, Florida, United States of America; Instituto Butantan, Brazil

## Abstract

**Background:**

The ascarosides form a family of small molecules that have been isolated from cultures of the nematode *Caenorhabditis elegans*. They are often referred to as “dauer pheromones” because most of them induce formation of long-lived and highly stress resistant dauer larvae. More recent studies have shown that ascarosides serve additional functions as social signals and mating pheromones. Thus, ascarosides have multiple functions. Until now, it has been generally assumed that ascarosides are constitutively expressed during nematode development.

**Methodology/Principal Findings:**

Cultures of *C. elegans* were developmentally synchronized on controlled diets. Ascarosides released into the media, as well as stored internally, were quantified by LC/MS. We found that ascaroside biosynthesis and release were strongly dependent on developmental stage and diet. The male attracting pheromone was verified to be a blend of at least four ascarosides, and peak production of the two most potent mating pheromone components, ascr#3 and asc#8 immediately preceded or coincided with the temporal window for mating. The concentration of ascr#2 increased under starvation conditions and peaked during dauer formation, strongly supporting ascr#2 as the main population density signal (dauer pheromone). After dauer formation, ascaroside production largely ceased and dauer larvae did not release any ascarosides. These findings show that both total ascaroside production and the relative proportions of individual ascarosides strongly correlate with these compounds' stage-specific biological functions.

**Conclusions/Significance:**

Ascaroside expression changes with development and environmental conditions. This is consistent with multiple functions of these signaling molecules. Knowledge of such differential regulation will make it possible to associate ascaroside production to gene expression profiles (transcript, protein or enzyme activity) and help to determine genetic pathways that control ascaroside biosynthesis. In conjunction with findings from previous studies, our results show that the pheromone system of *C. elegans* mimics that of insects in many ways, suggesting that pheromone signaling in *C. elegans* may exhibit functional homology also at the sensory level. In addition, our results provide a strong foundation for future behavioral modeling studies.

## Introduction

**Figure 1:**
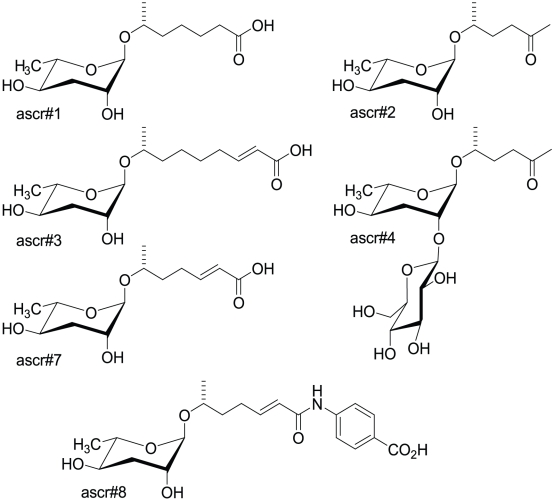
Ascarosides monitored in the study. Ascarosides consists of the sugar ascarylose linked to a fatty-acid-derived side chain that may contain a variety of functional groups.

*Caenorhabditis elegans*, a free-living roundworm (nematode) found in compost and rotting fruit [1], develops from fertilized eggs to adult animals in 3.5 days under optimal conditions of food and worm density. Under stressful conditions with low food, high nematode density, or high temperatures, L1 larvae progress into a developmentally arrested dauer stage [2], [3], [4] that is specialized for dispersal and can subsist for several months without food [2], [3], [4]. Entry into the dauer stage is known to be triggered by a dauer pheromone [3] and recently several different ascaroside pheromones have been identified as components of this pheromone [5], [6], [7]. Ascarosides (Figure 1) consist of the sugar ascarylose linked with a variety of fatty-acid-like side chains and, occasionally, other functional groups. Dauer forming activity typically occurs at nM to µM concentrations of ascarosides [6], [7], [8]. However, at pM concentrations a synergistic blend of ascarosides induces male-specific attraction to hermaphrodites [8], [9], [10].

Because dauer activity was found to be constitutive [3], the dauer pheromone has been assumed to be continuously expressed throughout development of the nematode [11]. Despite the identification of a number of new ascarosides and the increasing appreciation of their multiple functions in *C. elegans*, dependence of their expression on developmental stages has not been studied and the influence of environmental conditions on ascaroside production is poorly understood. Here we demonstrate that both relative and absolute amounts of ascarosides released to the environment, as well as unreleased ascarosides within the worms, are highly dependent on developmental stage and culturing conditions.

## Results

### Ascaroside release is regulated by development

**Figure 2:**
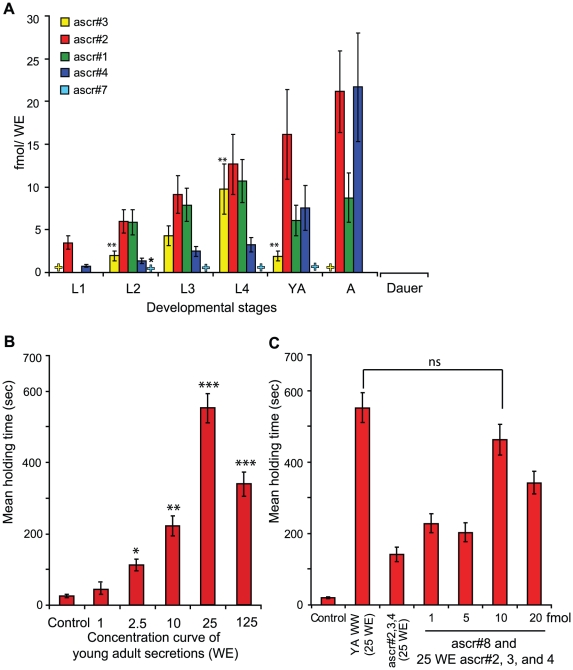
Ascaroside secretion of *C. elegans* at different developmental stages. (A) Concentrations of ascr#1, ascr#2, ascr#3, and ascr#4 in WW samples during development. One WE is defined as the amount of material released from one worm within 1 h. L1, L2, L3 and L4, larval stages; YA, young adult; A, adults with 10–15 eggs. For the columns labeled “Dauer”, 7 day old (163 h) dauer larvae were used. The figure represents the mean of four experiments and error bars, s.e.m. **P<0.05 (L4 to L2 and YA), unpaired *t*-test. +, detectable but not quantifiable and +*emphasize that ascr#7 is only quantifiable in L2 WW (0.5±0.08 fmol/WE). The ascr#4 concentrations were estimated using the ascr#2 concentration curve due to lack of synthetic ascr#4. Ascr#1 concentrations were estimated using its unsaturated analog, ascr#7, as a standard. (B) Concentration curve for mating activity of YA WW, n≥30 worms for each histogram. *P<0.05, **P<0.01, *** P<0.0001, using one-factor ANOVA with Dunnett's post-test. Error bars are s.e.m. (C) Reconstitution of male attraction activity with synthetic pheromone blend, n≥30 animals for each histogram. Based on LC-MS quantification, the amounts of the three main pheromone components in 25 WE were 537 fmol for ascr#2, 55 fmol for ascr#3 and 789 fmol for ascr#4. n≥30 animals for each histogram. However, the full synthetic blend consisted of ascr#2, ascr#3, ascr#4 and ascr#8. There was no significant difference in mating activity between YA WW (25 W.E.) and the synthetic blend when 10 fmol of ascr#8 was added to the 25 WE for ascr#2, ascr#3, and ascr#4, P<0.05, unpaired *t*-test. Error bars are s.e.m.

Hermaphrodite *C. elegans* (N2) were reared in liquid culture and developmentally synchronized as described earlier [8], [12], [13]. At each developmental stage worms were collected, cleaned and incubated in water for 1 h. The resulting worm water (WW) samples, only containing stage-specific secreted metabolites [8], were analyzed for all currently identified ascarosides, ascr#1, ascr#2, ascr#3, ascr#4, ascr#5, ascr#6, ascr#7 and ascr#8 [14] by liquid chromatography-mass spectrometry (LC-MS). Quantifications were based on synthetic standards [6], [10]. In this analysis, only ascr#1, ascr#2, ascr#3, ascr#4, and ascr#7 were present in detectable and quantifiable quantities from L1 to adults with eggs, whereas none of the known ascarosides were detected in WW of dauer larvae (Figure 1, Figure 2A, and Figure S1). The total ascaroside content increased with development (Figure 2A); in addition, the relative concentrations of the individual ascarosides showed stage-dependent variation. For example, the concentration of ascr#3 increased from L1 to L4 larvae (p<0.05 unpaired t-test, compare to L2 and young adult (YA)), peaking at ∼10 fmol/WE and then decreased at the YA and adult stages. Ascr#7 content peaked at the L2 stage (0.5±0.08 fmol/WE; where WE is defined as the material released from 1 worm in 1 h) with a decrease to very small (non quantifiable) amounts in L3, L4, and YA samples. Ascr#7 was not detectable in WW from L1 larvae and adults with eggs (Figure 2A). These data clearly show that ascr#1, ascr#2, ascr#3, ascr#4, and ascr#7 are differentially expressed during *C. elegans* development.

To rule out the possibility that the ascarosides detected in WW preparations were the result of hypo-osmotic shock, batches of YA worms were split into two samples, one was incubated in water and the other in M9 buffer. After 1 h the nematodes were removed and the supernatant analyzed by LC/MS. The analyses revealed that the total ascaroside concentrations in the M9 sample were higher than in water; however, the relative proportions of the most abundant ascarosides, ascr#2 and ascr#4, were similar (Figure S2).

### The male attracting pheromone is composed of at least four ascarosides

As shown previously, both ascr#3 and ascr#8 attract male *C. elegans* when tested at high concentrations (1 pmol) [8], [10], and stronger synergistic attraction was achieved by using 20 fmol of ascr#3 in combination with ascr#2 and ascr#4 [8]. This synthetic blend was still less active than a natural YA WW preparation with similar ascaroside concentrations. Following the identification of ascr#8 via NMR-spectroscopic methods from large liquid cultures [10], less than 1 fmol/WE of ascr#8 was found by LC-MS in YA WW (Figure S1 B). Maximal male attraction was found using an amount of 25 WE per assay (Figure 2B). By adding 10 fmol of ascr#8 to 25 WE of the synthetic blend (Figure 2C), the mating attraction could be fully restored to YA WW level (p<0.14 *t*-test unpaired). This confirms that the male attracting pheromone is a synergistic blend of at least four ascarosides.

### Ascaroside release is regulated by diet

**Figure 3:**
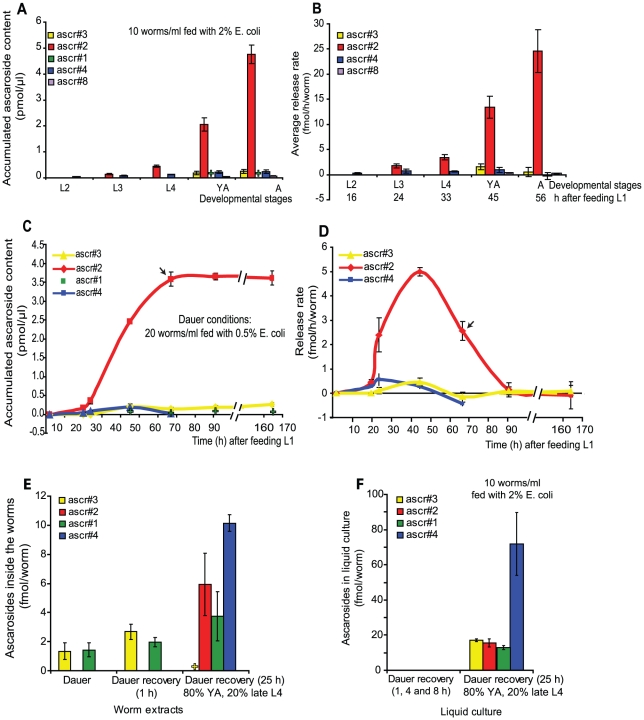
Changes in *C. elegans* ascaroside production during development in standard and dauer forming liquid cultures. Ascaroside concentrations were determined via LC-MS analyses in positive ion mode for ascr#2 and ascr#4 and negative ion mode for ascr#1, ascr#3, ascr#7 and ascr#8. (A) Concentrations of ascr#2, ascr#3, ascr#4, and ascr#8 in media samples taken from synchronized standard liquid cultures of L2, L3, L4, YA and adults with 10–15 eggs in standard liquid cultures (10,000 worms/ml fed with 2% *E. coli*). The figure represents the mean of four experiments and error bars, s.e.m. (B) Average release rate calculated as changes in concentration (after each life stage transition subtracting the concentrations determined for the previous life stage) divided by the worm density and the number of hours since previous sampling. (C) Concentration of ascr#2, ascr#3, and ascr#4 during dauer forming liquid cultures (20,000 worms/ml fed with 0.5% *E. coli*). Ascr#3 values were calculated from LC-MS analysis run positive ion mode. Ascr#1 was detectable but not quantifiable. The figure represents the mean of four experiments and error bars, s.e.m. (D) Release rate, calculated by subtracting the concentrations determined for the previous time point and divided by the hours and worm density. Arrow at 67 h marks the time point at which 60% of the worms were SDS resistant. ND, not detected. (E) Ascaroside content of whole-body extracts of dauer and dauer recovered worm. Data shown are mean values from two experiments for dauer worm extracts and three experiments for dauer recovery extracts. (F) Ascaroside content of liquid culture of dauer recovered worms. The figure represents the mean of four experiments and error bars, s.e.m. +, detectable but not quantifiable.

WW collection from carefully synchronized worms is a useful method for determining developmental stage-dependent changes but places the worms in an environment without food or accumulated pheromones which could influence the release of ascarosides. Therefore, we sampled the ascaroside content of synchronized worms in liquid culture, complete with bacteria, at time points corresponding to different developmental stages. Worms were grown at moderate worm density and abundant food (10,000 worms/ml fed with 2% *E. coli* (HB101)) as well as typical dauer-forming conditions with high worm density and low food (20,000 worms/ml fed with 0.5% *E. coli* (HB101)). Only ascr#1, ascr#2, ascr#3, ascr#4 and ascr#8 were detectable and quantifiable in the standard worm liquid cultures (Figure 3A). In the dauer-forming liquid cultures, asrc#1, ascr#2, ascr#3 and ascr#4 were detectable, but only ascr#2, ascr#3 and ascr#4 were quantifiable (Figure 3C). The WW preparation (Figure 2A) is a one-hour “snap shot” of the ascarosides released by nematodes at defined developmental stages after they have been transferred to water. In contrast, the liquid co-culture data represents a series of samplings of an accumulative exogenous ascaroside profile. Therefore, the liquid co-culture analyses data (Figure 3A, B, C and D) are presented in two ways: First, the data are presented as the accumulative ascaroside concentrations in the media, analyzed either at time points representative of each developmental stage (Figure 3A) or at fixed time intervals (Figure 3C). Second, the data are presented as average release/h for each transition between developmental stages in the normal growth media (Figure 3B) and as release/h for the transition to dauer at fixed time points (Figure 3D). For all developmental stages, ascr#2 was the major ascaroside released in liquid co-cultures while asc#4 was a minor constituent (Figure 3A, B, C and D). This is consistent with previous studies [10], but different from our WW preparations where the ascr#2 and ascr#4 concentrations are similar (Figure 2A). In the well-fed liquid cultures, the largest amount of ascr#3 and ascr#8 were released by YA and adults (Figure 3A and B). In contrast, in WW the largest amount of ascr#3 was collected from L4 stage which might be a result of the longer preparation time needed for WW sample collection (3 to 4 h between culture sampling and WW extraction) compared to the direct sampling of the liquid culture. Ascr#8 was detectable only in trace amount in WW from YA worms. Surprisingly, in dauer-forming liquid cultures the maximal ascaroside production appears to coincide with the transition into dauer when ascr#2, asc#3 and ascr#4 are produced in a burst between 20 and 45 h, followed by a rapid drop-off in ascaroside production as the larvae enter dauer (Figure 3C and D). Jeong et al [15] reported that synchronized L1 larvae, when treated with ascr#1 and incubated at 25°C, started forming dauers at 48 h, which coincides with the large increase in ascr#2 release we observed. Moreover, there was no additional change in ascaroside content in the dauer-forming culture after 70 h which is consistent with Jeong et al [15] where the dauer formation was complete at 72 h. These results strongly suggest that dauers do not release ascarosides, consistent with the observed lack of detectable amounts of ascarosides in dauer WW (Figure 2A). Ascaroside production in dauer larvae was further investigated by analyzing whole body extracts of the dauer worms which showed that worm interior contained small, but significant amounts of ascr#1 and ascr#3 (Figure 3E), though none of the other ascarosides could be detected. This indicates that although ascarosides are constitutively present they are not released by dauer worms.

### Dauer recovered worms have a different ascaroside profile

The initiation of ascaroside production and secretion during and after dauer recovery was studied by transferring the dauers to rich recovery media (10,000 worms/ml with 2% *E. coli*). During the first 8 h following transfer, ascr#1 and ascr#3 continued to be the only ascarosides detectable inside the worms with no ascarosides detected in the liquid culture (Figure 3E and F). However, after 25 h, a time at which the majority of the nematodes had developed into young adults, large amounts of ascr#1, ascr#2, ascr#3 and a strongly dominating ascr#4 had been released into the liquid culture (Figure 3E). Furthermore, the amounts of ascr#2 and ascr#4 inside the worms had significantly increased at 25 h (Figure 3F).

## Discussion

This investigation shows that release and production of ascarosides by *C. elegans* is highly dependent on developmental and environmental conditions. During growth conditions favoring reproductive development, the concentrations of the dominating ascarosides ascr#2 and ascr#4 gradually increase with development, but remain relatively low during the early larval stages L2 and L3 (Figure 2A, 3A and 3B). In contrast, under dauer inducing conditions (Figure 3C and D), large quantities of ascr#2 are produced immediately prior to entering dauer, followed by complete cessation of ascaroside secretion after entering dauer. Consistent with our results, Joo et al [16] also identified ascr#2 as the major ascaroside in their dauer forming cultures and only small amounts of ascr#3 and ascr#1. Interestingly, ascr#2 is the most strongly dauer-inducing compound among the known ascarosides, suggesting that the burst of ascr#2 produced by pre-dauer worms constitutes a population density signal. The only ascarosides found within dauer larvae are small quantities of ascr#1 and ascr#3, which do not appear to be released into the media. Previous studies by Joo et al [16] also reported the presence of ascr#1 and ascr#3 in worm extracts from dauer cultures but, in contrast to our findings, Joo et al. also found ascr#2. However, preparation of dauers from a multi-generation mixed-stage culture as used by Joo et al. is likely to result in samples containing not only dauer larvae, but also smaller quantities of other life stages. Therefore, it is possible that the detection of ascr#2 in dauer worm samples by Joo et al. was due to contamination with non-dauer worms. We also found that the ratio of ascr#2/ascr#4 depends on the life history of the worms where the high ratio of ascr#2/ascr#4 was secreted by young adults from the standard liquid culture (Figure 3A and C). A much lower ascr#2/ascr#4 ratio was observed for young adults recovered from dauer (Figure 3F). Recently, a similar phenomena was observed at the gene expression level, where animals that passed through dauer larval stage revealed unique gene expression profiles compare to adult animals that did not [17]. The finding that most ascr#4 is produced by YA worms recovered from dauer may indicate that the ascr#2/ascr#4 ratio plays a role in signaling specific environmental conditions. The observed expression pattern (Figure 2A and 3A) of ascr#3 and ascr#8 also correlates to their biological function where their production maximum immediately precedes or coincides with the temporal window for mating.

We have shown that both relative and absolute expression of ascarosides are complex and change with development as well as in response to multiple environmental factors. This is consistent with multiple functions of ascarosides and demonstrates that various blends play important roles converting environmental conditions into appropriate behavioral responses.

### Future studies

The ability to control the previously unknown differential regulation of ascarosides will make it possible to associate ascaroside production to gene expression profiles (transcript profile [15], [17], protein levels or enzyme activity) and thus help to dissect the biosynthetic pathways for the structurally diverse ascarosides. It will also help to elucidate signaling processes involved in regulation of ascaroside synthesis and release, which in turn affect the behavior of nematodes. Additionally, since the complexity of the pheromone system of this nematode in many ways mimics that of insects, we suggest that *C. elegans* is functionally homologous for studies of pheromone perception at the sensory level. Finally, this study also provides a strong foundation for future behavioral modeling studies.

## Materials and Methods

### Nematode strains and growth conditions

Synchronized *C. elegans* (N2 Bristol) were co-cultured with 2% *E. coli* (strain HB101) at a worm density of ∼10,000 worms/ml for standard liquid culture or with 0.5% *E. coli* (strain HB101) with a worm density of ∼20,000 worms/ml for dauer forming liquid culture at 22°C shaking at 250 rpm in S-complete medium [8].

### Collecting ascarosides in water

Synchronized worms at the desired life stages except for L1 were harvested from liquid culture and separated from the medium and *E. coli* by gravity and subsequently rinsed three times over a nylon filter. They were then allowed to digest bacteria in their gut for 30 min in M9 buffer, rinsed three times with water, and incubated in water for 1 h at density of ∼30,000 worms/ml, as previously described [8], [12], [13], [18]. Collecting secretions from L1: Eggs were hatched in M9 buffer for 24 h. The synchronized L1 were washed with sterile water three times and incubated 1 h in water.

### Collecting ascarosides in M9 buffer

The worms were grown and cleaned up as described above. After cleaning up their gut in M9 buffer, they were washed three times in double distilled water and divided into two batches; one half of the worms were placed in M9 buffer and the other half was placed in water for ascaroside collection for 1 h.

### Collecting ascarosides in worm liquid cultures

For standard liquid culture, synchronized *C. elegans* (N2 Bristol) were incubated at a worm density of ∼10,000 worms/ml in S-complete medium with 2% *E. coli* (strain HB101) at 22°C with shaking at 250 rpm [8]. When the nematodes reached the desired life stage, 10 ml of liquid culture was taken for ascaroside analysis. L1 worms were prepared by letting eggs hatch in M9 buffer for 24 h. Sampling time after transfer to feeding medium was 16 h for L2, 24 h for L3, 33 h for L4, 45 h for YA, and 56 h for adult (A) stage worms. Worms were identified as L4, YA and A after inspection under a microscope. For dauer forming liquid cultures, synchronized *C. elegans* (N2 Bristol) were incubated at a worm density of ∼20,000 worms/ml in S-complete medium with 0.5% *E. coli* (HB101) at 22°C at 250 rpm. Samples were taken 2, 20, 24, 67, 91, 115, 139 and 163 h after starting the experiment. For sample collection, nematodes were separated by gravity for 10 min from the medium followed by centrifugation at 1500 rpm for 3 min. Supernatant was separated and centrifuged again at 4400 rpm for 5 min. Last centrifugation of the supernatant was done at 6000 rpm for 5 min and filter sterilized (0.22 micron filters) and stored in −80°C.

### SDS test for dauer larvae

Worms were washed three times in M9 buffer, placed in 1% SDS for 15 min [19], and then checked for survival. Worms that were alive were scored as dauer.

### Ascaroside LC-MS analysis

One ml of worm water or 1 ml of worm liquid culture was lyophilized and resuspended in 100 µl of 50% MeOH. Insoluble materials were separated by centrifugation at 6,000 g for 5 min. Ascarosides were analyzed by thermo spray Liquid Chromatography and Mass Spectrometry (LC/MS) using a Thermo Finnigan LCQ Deca XP Max equipped with a polymer column PLRP-S (Varian, Inc) in the positive and negative ion mode (sheath gas flow 20 au, aux gas 5 au, spray voltage 5 kV and transfer line temperature of 280°C). Ten or 20 µl of samples were injected on a 5 µM PLRP-S column using a column oven temperature of 60°C and a flow rate of 1.0 ml/min with a gradient of solvent A (0.1% formic acid water) and solvent B (90% acetonitrile with 10 mM ammonium formate) from 90% solvent A and 10% solvent B for 2 min followed by a linear gradient to 95% B in 18 min and a 5 min return to the starting composition. Separate analyses were performed to quantify ascr#1, ascr#3 and ascr#8 content in negative ion mode and ascr#2, ascr#4 and ascr#7 in positive ion mode. Standard curves were prepared for all ascarosides using synthetic compounds prior to analyses and control samples were analyzed before and after the WW and liquid culture samples.

### Worm extracts

Dauers were produced in dauer forming liquid culture (20,000 worms/ml fed with 0.5% *E. coli*). At day 5 of the experiment, worms were treated with 1% SDS and the live worms harvested using sucrose and ficoll gradients [19]. The remaining dead worms were separated from the live dauer worms by plating, allowing the live worms to move away from debris of dead worms. The dead worms were removed and the live worms collected by washing the plates with M9 buffer. Twenty thousand dauer worms were washed three times with water and placed in 70% ethanol and subsequently frozen at −80°C freezer for preparation of dauer worm extracts.

Dauer worms were kept at 4°C for 3 days and fed with 2% HB101 with a density of 10,000 worms/ml for dauer recovery. Thirty thousand dauer recovered worms (1 and 25 h after feeding dauers) were rinsed three times with water and placed in 1 ml of 70% MeOH or EtOH and stored in −80°C until extraction. One g of ceramic zirconium beads (1.25 mm) (ZIRMIL) was added to the nematodes. Then nematodes were ground five times for 37 sec using a Precellys24 (http://www.precellys.com) homogenizer. The worms were checked under a microscope to verify complete homogenization. The worm debris was removed by centrifugation at 6,000 g for 5 min. Eight hundred µl of supernatant was removed, dried using a stream of nitrogen, and resuspended in 80 µl of 50% MeOH. Twenty µl of the resulting worm extract was analyzed by LC-MS in positive or negative ion mode to determine the ascr#1, ascr#2, ascr#3, ascr#4, ascr#7 and ascr#8 contents.

### Male attraction assays

The bioassays with the ascarosides were done as previously described [8].

### Statistical analysis for attraction assays (Figure 2B and C)

Since all attraction assays were conducted along with controls, we used one-factor ANOVA followed by Dunnett's post-test to compare differences between control and ascaroside activity.

## Supporting Information

Figure S1 **LC-MS profile of ascarosides of YA used for the male attraction assay.** (A) ascr#2, ascr#3 and ascr#4 and their m/z ratios. (B) MSMS analysis of asc#8 M+1 ion at m/z 264 using isolation with of 2 m/z, normalized collision energy of 35%, activation Q 0.200 and activation time 30 ms giving a dominating and characteristic M+1-18 fragment at m/z 246. Upper panel is for the RIC for ascr#8 standard (0.3 ng/µl), young adult WW, young adult WW spiked with ascr#8 (0.3 ng/µl) and lower panel is solvent control profile showing no corresponding m/z 246 fragment.
(EPS)

Figure S2 **Ascaroside concentrations in WW samples using M9 buffer or Water.** Ascarosides collected from young adults in water and M9 buffer from young adults were analyzed in positive and negative ion mode by LC-MS. The data represent the mean of two experiments. Ascr#2, ascr#3, and ascr#4 were detectable, but only ascr#2 and ascr#4 were quantifiable amounts in both water and M9 buffer. Error bars are standard deviation.
(EPS)
